# Addition of HIV self-test kits to partner notification services to increase HIV testing of male partners of pregnant women in Zambia: two parallel randomised trials

**DOI:** 10.1016/S2214-109X(21)00393-4

**Published:** 2021-11-01

**Authors:** Wilbroad Mutale, Kellie Freeborn, Lauren A Graybill, Mildred M Lusaka, Katie R Mollan, Oliver Mweemba, Margaret Kasaro, Rose Lungu, Andrew Kumwenda, Friday Saidi, Kimberly A Powers, Suzanne Maman, Nora E Rosenberg, Benjamin H Chi

**Affiliations:** School of Public Health, University of Zambia, Lusaka, Zambia; School of Medicine, University of North Carolina at Chapel Hill, Chapel Hill, NC, USA; Gillings School of Global Public Health, University of North Carolina at Chapel Hill, Chapel Hill, NC, USA; UNC Global Projects - Zambia, Lusaka, Zambia; School of Medicine, University of North Carolina at Chapel Hill, Chapel Hill, NC, USA; Gillings School of Global Public Health, University of North Carolina at Chapel Hill, Chapel Hill, NC, USA; School of Public Health, University of Zambia, Lusaka, Zambia; UNC Global Projects - Zambia, Lusaka, Zambia; UNC Global Projects - Zambia, Lusaka, Zambia; School of Medicine, University of Zambia, Lusaka, Zambia; UNC Project Malawi, Lilongwe, Malawi; Gillings School of Global Public Health, University of North Carolina at Chapel Hill, Chapel Hill, NC, USA; Gillings School of Global Public Health, University of North Carolina at Chapel Hill, Chapel Hill, NC, USA; Gillings School of Global Public Health, University of North Carolina at Chapel Hill, Chapel Hill, NC, USA; School of Medicine, University of North Carolina at Chapel Hill, Chapel Hill, NC, USA

## Abstract

**Background:**

Testing men for HIV during their partner’s pregnancy can guide couples-based HIV prevention and treatment, but testing rates remain low. We investigated a combination approach, using evidence-based strategies, to increase HIV testing in male partners of HIV-positive and HIV-negative pregnant women.

**Methods:**

We did two parallel, unmasked randomised trials, enrolling pregnant women who had an HIV-positive test result documented in their antenatal record (trial 1) and women who had an HIV-negative test result documented in their antenatal record (trial 2) from an antenatal setting in Lusaka, Zambia. Women in both trials were randomly assigned (1:1) to the intervention or control groups using permuted block randomisation. The control groups received partner notification services only, including an adapted version for women who were HIV-negative; the intervention groups additionally received targeted education on the use of oral HIV self-test kits for their partners, along with up to five oral HIV self-test kits. At the 30 day follow-up we collected information from pregnant women about their primary male partner’s HIV testing in the previous 30 days at health-care facilities, at home, or at any other facility. Our primary outcome was reported male partner testing at a health facility within 30 days following randomisation using a complete-case approach. Women also reported male partner HIV testing of any kind (including self-testing at home) that occurred within 30 days. Randomisation groups were compared via probability difference with a corresponding Wald-based 95% CI. The trial is registered at ClinicalTrials.gov (NCT04124536) and all enrolment and follow-up has been completed.

**Findings:**

From Oct 28, 2019, to May 26, 2020, 116 women who were HIV-positive (trial 1) and 210 women who were HIV-negative (trial 2) were enrolled and randomly assigned to study groups. Retention at 30 days was 100 (86%) in trial 1 and 200 (95%) in trial 2. Women in the intervention group were less likely to report facility-based male partner HIV testing in trial 1 (3 [6%] of 47 *vs* 15 [28%] of 53, estimated probability difference −21·9% [95% CI −35·9 to −7·9%]) and trial 2 (3 [3%] of 102 *vs* 33 [34%] of 98, estimated probability difference −30·7% [95% CI −40·6 to −20·8]). However, reported male partner HIV testing of any kind was higher in the intervention group than in the control group in trial 1 (36 [77%] of 47 *vs* 19 [36%] of 53, estimated probability difference 40·7% [95% CI 23·0 to 58·4%]) and trial 2 (80 [78%] of 102 *vs* 54 [55%] of 98, estimated probability difference 23·3% [95% CI 10·7 to 36·0%]) due to increased use of HIV self-testing. Overall, 14 male partners tested HIV-positive. Across the two trials, three cases of intimate partner violence were reported (two in the control groups and one in the intervention groups).

**Interpretation:**

Our combination approach increased overall HIV testing in male partners of pregnant women but reduced the proportion of men who sought follow-up facility-based testing. This combination approach might reduce linkages to health care, including for HIV prevention, and should be considered in the design of comprehensive HIV programmes.

**Funding:**

National Institutes of Health.

## Introduction

Important global achievements have been made in the prevention of mother-to-child (PMTCT) HIV transmission. In sub-Saharan Africa, where the burden of HIV is greatest, involving male partners in health services improves antenatal care attendance, PMTCT programme uptake, and infant survival.^1-3^ Male partner HIV testing is an essential component to this engagement and can further optimise family-based HIV prevention, care, and treatment. Although the uptake of male partner HIV testing has remained low in many programme settings,^4^ several new interventions show promise, including assisted partner notification services, home-based testing, and HIV self-testing.^5,6^ However, these individual approaches alone might not be enough to broaden coverage to meet the ambitious HIV testing targets set by the global HIV/AIDS community. The combination of different evidence-based strategies could further enhance HIV testing uptake but, to date, there are little data to support combination strategies.

We developed a couples-based framework for HIV prevention and treatment in antenatal settings.^7^ Using this framework in the setting of comprehensive HIV services, mathematical modelling done by our group showed that small to moderate increases in male partner HIV testing might lead to substantial reductions in horizontal and vertical HIV transmission.^8^ Public health strategies, including index testing and active case finding,^9^ typically focus on pregnant women who test HIV-positive to efficiently identify male partners with undiagnosed HIV. However, such approaches can lead to missed opportunities because pregnant women who initially test HIV-negative, but remain at elevated risk for acquiring HIV, are overlooked. Universal health services for the male partners of all pregnant women (ie, status-neutral approaches^10^) can increase acceptability, reduce stigma, and broaden overall reach and effect.

Here, we report the primary findings of two parallel randomised trials designed to address gaps in male partner HIV testing: one in HIV-positive pregnant women and one in HIV-negative pregnant women. Our integrated intervention included two strategies recommended by WHO: assisted partner notification services (ie, voluntary and health-care provider-supported identification and tracing of partners for HIV testing) and secondary distribution of HIV self-test kits (ie, provision of HIV self-test kits to the index pregnant women for use by their partners and themselves).^11^

## Methods

### Study design and participants

We did two parallel, unmasked, individual-level, randomised controlled trials. Participating women were recruited at the Chipata Level 1 Hospital, a government health facility in Zambia’s capital city of Lusaka. Serving a catchment population of over 100 000, this busy facility has an antenatal HIV prevalence of 16%. Eligible participants met the following criteria: 18 years or older, pregnant and seeking antenatal care at the time of enrolment, documented HIV status in their antenatal record, at least one current male sex partner, willingness to provide her contact information, ability and willingness to provide informed consent, and willingness to adhere to study procedures. Women who had already tested for HIV with their partner (eg, they received couples’ HIV testing) during the current pregnancy were excluded, as were those who expressed concerns about intimate partner violence or social harms at screening. Eligible women were offered enrolment into one of two trials according to their HIV status. In trial 1, we enrolled pregnant women who had an HIV-positive test result in their antenatal record; in trial 2, we enrolled women who had an HIV-negative test result in their antenatal record within the past 3 months during the current pregnancy. In each trial, participants were randomly assigned to either the control or intervention group.

All study participants were fully informed of the study procedures and provided informed written consent. The study protocol was approved by research ethics committees or institutional review boards at the University of Zambia (Lusaka, Zambia) and the University of North Carolina (Chapel Hill, NC, USA). We also obtained in-country approvals from the Zambia National Health Research Authority and the Lusaka District Health Office before the study began.

### Randomisation

Women in both trials were randomly assigned (1:1) to the intervention or control groups using permuted block randomisation. The random allocation sequences with block sizes of 2, 4, and 6 for both trials were generated by the study biostatistician (KRM) using SAS version 9.4 and were kept confidential from the study team during study conduct. Randomisation assignments were placed in sealed, opaque numbered envelopes by personnel in Lusaka who were not directly involved with participant enrolment. At the enrolment visit, study staff would open the next envelope in sequence. These allocations were documented and monitored for quality assurance according to standard practice.^12^

### Procedures

Our team worked with community partners and hospital staff to provide information about the study and facilitate recruitment. In a prescreening stage, pregnant women who were interested in the study were first asked if they had been tested for HIV with their primary male partners during the current pregnancy. For women who had not undergone couple HIV testing with their partner in the current pregnancy, a full screening questionnaire was completed. All participants provided written informed consent in either English, Bemba, or Nyanja. At enrolment, participants answered questions about their sociodemographic characteristics, obstetrical history, and sexual health. They also provided information about individual and primary male partner HIV testing history, current HIV treatment and prevention measures, and history of intimate partner violence. Regardless of study group, participants in both trials were offered four options for male partner HIV testing: client self-referral (the woman is encouraged to disclose her HIV status to her male partner and suggest they are tested for HIV), provider contract referral (the woman enters into a contract with the health-care provider to suggest HIV testing to their male partner within a set time period and, if testing does not occur, she gives trained providers permission to contact the partner directly and offer testing services), provider referral (with consent of the woman, a trained provider confidentially contacts the partner directly and offers HIV testing), and dual referral (a trained provider accompanies the woman, provides support during the HIV status disclosure process, and offers partner HIV testing).^13^ These options are already provided to pregnant women living with HIV in Zambia, in a package that is generally referred to as assisted partner notification services (shortened to partner notification services in the remainder of this report).^14^ In line with our status-neutral approach, we also adapted this strategy for HIV-negative pregnant women; we offered the same four options for male partner testing, but tailored the counselling messages to focus on HIV prevention in the context of the woman’s HIV-negative status.

In trial 1 (women who were HIV-positive), all participants informed the study staff of the partner notification approach they had previously selected as part of routine antenatal care. Women in trial 2 (women who were HIV-negative) were educated about the similar partner notification options and asked to select their preferred approach, which was then implemented by our study staff. In both trials, participants randomly allocated to the intervention groups additionally received targeted education on the use of oral HIV self-test kits for their partners (and for themselves if desired). Written instructions for the testing procedures were provided in English, Bemba, and Nyanja, using materials approved by the Zambia Ministry of Health. Up to five oral HIV self-test kits (OraQuick, Orasure Technologies, Bethlehem, PA, USA) per participant were offered as part of the intervention. HIV testing at a health facility by a healthcare provider was recommended to all study participants regardless of study group allocation. This included confirmatory HIV testing at a healthcare facility for those who first tested at their home or in the community, regardless of their HIV test result. This advice differed slightly from local HIV self-testing guidelines, which, similar to WHO recommendations at the time,^13^ did not recommend confirmatory testing for non-reactive HIV self-test kits in low-risk individuals.^14^ However, in this study, facility-based HIV testing was viewed as an important entry point to HIV services for male partners and a key outcome of interest.

All participants were scheduled to return for a follow-up visit approximately 30 days from enrolment. At the follow-up visit, we collected information from pregnant women about primary male partner HIV testing, including the date, modality, and venue. We screened for intimate partner violence through a nine-item questionnaire previously adapted from the Kenya Demographic and Health Survey.^15^ We also asked about social harms related to trial participation, with questions adapted from previous studies in Malawi.^16^ Women who reported intimate partner violence or social harms at their follow-up visit were counselled. As appropriate, our staff provided referrals to the gender-based violence command centre, located on hospital grounds. Information about other adverse events were collected via interviews and medical record reviews.

With the emergence of COVID-19, we made modifications to our follow-up protocols from March, 2020 onward. Although our main outcome remained the same (ie, reported primary male partner HIV testing within the first 30 days), we extended the window period for data collection. To promote physical distancing and to limit in-person contact, we also offered telephone interviews at follow-up for those unable to travel safely to the study clinic.

### Outcomes

Our main outcome was the proportion of primary male partners reported to have tested for HIV at a health facility within 30 days of participant enrolment. Specifying testing at a health-care facility emphasises the importance of HIV testing by trained personnel and the need for engaging with HIV services. The short window (ie, 30 days) reflects the urgency of male partner HIV testing in the context of PMTCT, particularly for settings like Zambia where antenatal care typically begins later in pregnancy. A prespecified outcome was the proportion of primary male partners reported to have HIV testing of any kind (including HIV self-testing in the household and other community-based venues) within 30 days of randomisation. This outcome was added on May 1, 2020, before study completion. Similar to other studies,^15,17^ to measure male partner HIV testing outcomes, we relied on the female participant’s report. To be included in the numerator for either of the study outcomes, the reported testing event had to occur within 30 days of randomisation. Of those women whose main partner had been tested for HIV, we reported the results of the HIV test. We also described incident social harms, intimate partner violence, and other serious adverse events.

### Statistical analysis

This study was designed a priori to assess acceptability, feasibility, and early effectiveness of partner HIV testing strategies. We calculated our sample size on the basis of large effect sizes in partner HIV testing outcomes. For each trial, we used a type I error rate of α=0·05 and two-sided CIs with no adjustment for multiplicity and anticipated 5% missing data and attrition for power calculations. In trial 1 (women who were HIV-positive), we assumed that 20% of male partners in the control group would be tested for HIV. We anticipated a 25 percentage point increase (ie, 20% *vs* 45%) in facility-based HIV testing rates with our intervention strategy. An enrolment target of 116 HIV-positive pregnant women (58 women per group) provided approximately 80% power to detect this anticipated difference. In trial 2 (women who were HIV-negative), we estimated that 10% of male partners in the control group would receive facility-based HIV testing. With the addition of HIV self-testing (as provided to the intervention group), we expected a 15 percentage point increase in facility-based partner HIV testing rates (ie, 10% *vs* 25%). Based on these assumptions, our sample size of 210 HIV-negative pregnant women (105 per group) provided approximately 80% power. Differences in anticipated baseline HIV testing and effect size for trials 1 and 2 reflect our underlying assumptions about facility-based testing. Male partners of women who were HIV-positive might be more likely to seek facility-based testing to confirm results and to engage with treatment or prevention services as needed, because of their higher risk for HIV, for example, than male partners of women who were HIV-negative. Conversely, because male partners of women who were HIV-negative are more likely to test HIV-negative themselves, we reasoned that they could be less likely to return for facility-based HIV testing than male partners of women who were HIV-positive.^18^

The two trial analyses were done separately, using a complete-case approach with the woman as the unit of analysis. Participants in each trial were analysed according to the exposure group to which they were randomly assigned. For the primary analysis, we compared the proportion of women reporting facility-based male partner HIV testing within 30 days of randomisation between the intervention and control groups using an estimated probability difference (analogous to a risk difference) and its corresponding Wald-based 95% CI. Using the same approach, we also estimated a probability difference to compare reported male partner HIV testing of any kind within 30 days of randomisation between study groups. We did a prespecified sensitivity analysis to estimate a probability difference (intervention–control) adjusted for partner age, age difference between the partner and study participant, travel time to the health facility, and partner HIV testing history by applying an augmented inverse probability weighted doubly robust method, with corresponding bias-corrected percentile bootstrapped 95% CIs.^19,20^ Additional pooled analyses of the two trials were done using direct standardisation to account for the 16% HIV prevalence within the study site’s catchment area. In an ancillary time-to-event analysis, we used Kaplan-Meier estimation to describe days from randomisation to reported partner HIV testing in the intervention and control groups of both trials. Women whose partner had not yet completed HIV testing had follow-up time right-censored at 30 days.

Women with no follow-up, or who presented after the visit window, were considered missing and excluded from the denominator for complete-case analyses. In prespecified sensitivity analyses we evaluated the effect of missing data by non-parametrically estimating best case and worst case bounds around our effect estimates.^21^ In these sensitivity analyses, all randomly assigned participants were included in the analysis. Best case bounds were constructed by assuming that all women missing outcome data in the intervention groups had a partner who tested within 30 days of randomisation and that every woman missing outcome data in the control groups did not have a partner who tested within 30 days of randomisation. The opposite assumptions were made when estimating the worst case bounds. Additionally, multiple imputation of missing partner HIV testing outcomes was done using fully conditional specification (discriminant function method) with 50 imputed datasets, separately for trials 1 and 2. Each imputation model included partner testing outcomes, randomisation group, the four baseline covariates in our adjusted analyses, and two-way interactions between randomisation group and each baseline covariate. Rubin’s rule was used to combine the results from the 50 imputed datasets.

An affirmative response to any question on either the social harms or intimate partner violence instruments administered at follow-up was counted as an incident social harm or intimate partner violence event. The number of women who reported social harms and intimate partner violence was summarised separately by randomisation group within each trial. All analyses were done using Windows SAS version 9.4. The trial is registered at ClinicalTrials.gov (NCT04124536).

### Role of the funding source

The funders of the study had no role in study design, data collection, data analysis, data interpretation, or writing of this paper.

## Results

**Figure 1:**
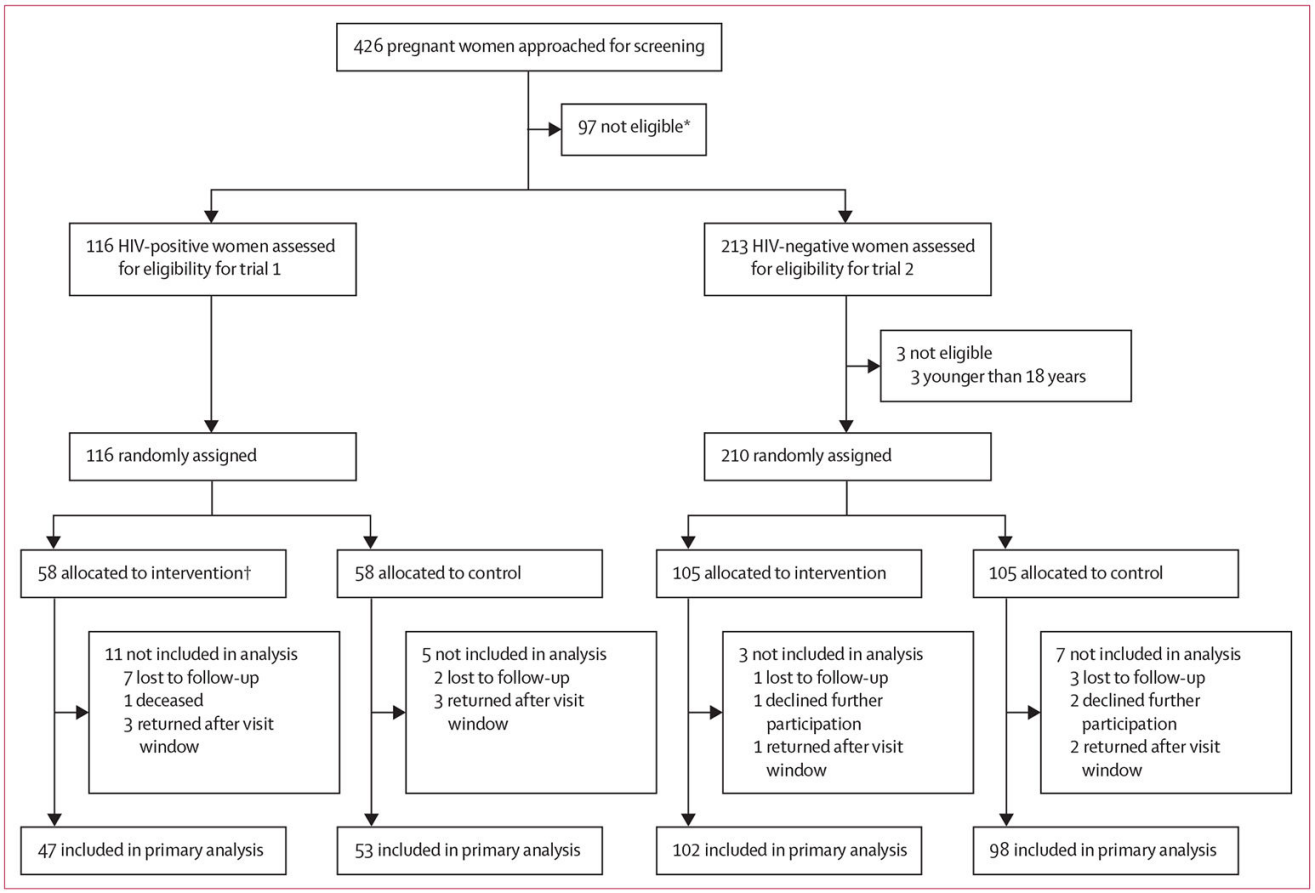
Trial profile *Already tested for HIV with partner during current pregnancy. †One participant did not receive the assigned intervention because she declined the HIV self-testing kit. This participant was included as exposed to the intervention in the primary analysis.

From Oct 28, 2019, to May 26, 2020, 426 pregnant women receiving antenatal care at the study site were approached about the study; 97 (23%) reported testing with their male partners during the current pregnancy and were excluded from further evaluation. The remaining 329 (77%) were assessed for full eligibility. Three HIV-negative women were found to be younger than 18 years and were also excluded. Of the remaining 326 women, 116 (36%) women who were HIV-positive were enrolled and randomly assigned in trial 1 (58 to the intervention group, 58 to control); 210 women who were HIV-negative were enrolled and randomly assigned in trial 2 (105 to the intervention group, 105 to control). The last study visit was completed on July 3, 2020. Overall, retention was high: 100 (86%) of 116 women who were HIV-positive and 200 (95%) of 210 women who were HIV-negative completed follow-up (figure 1). Key demographic, socioeconomic, and clinical characteristics are shown in table 1. At enrolment, women were asked to choose one of the four partner notification strategies. Nearly all women chose the client self-referral approach: 114 (98%) in trial 1 and 207 (99%) in trial 2. The remaining five women opted for the provider contract referral, with relative balance in the trials (two in trial 1 and three in trial 2) and randomisation groups (two in the intervention groups and three in the control groups).

**Figure 2:**
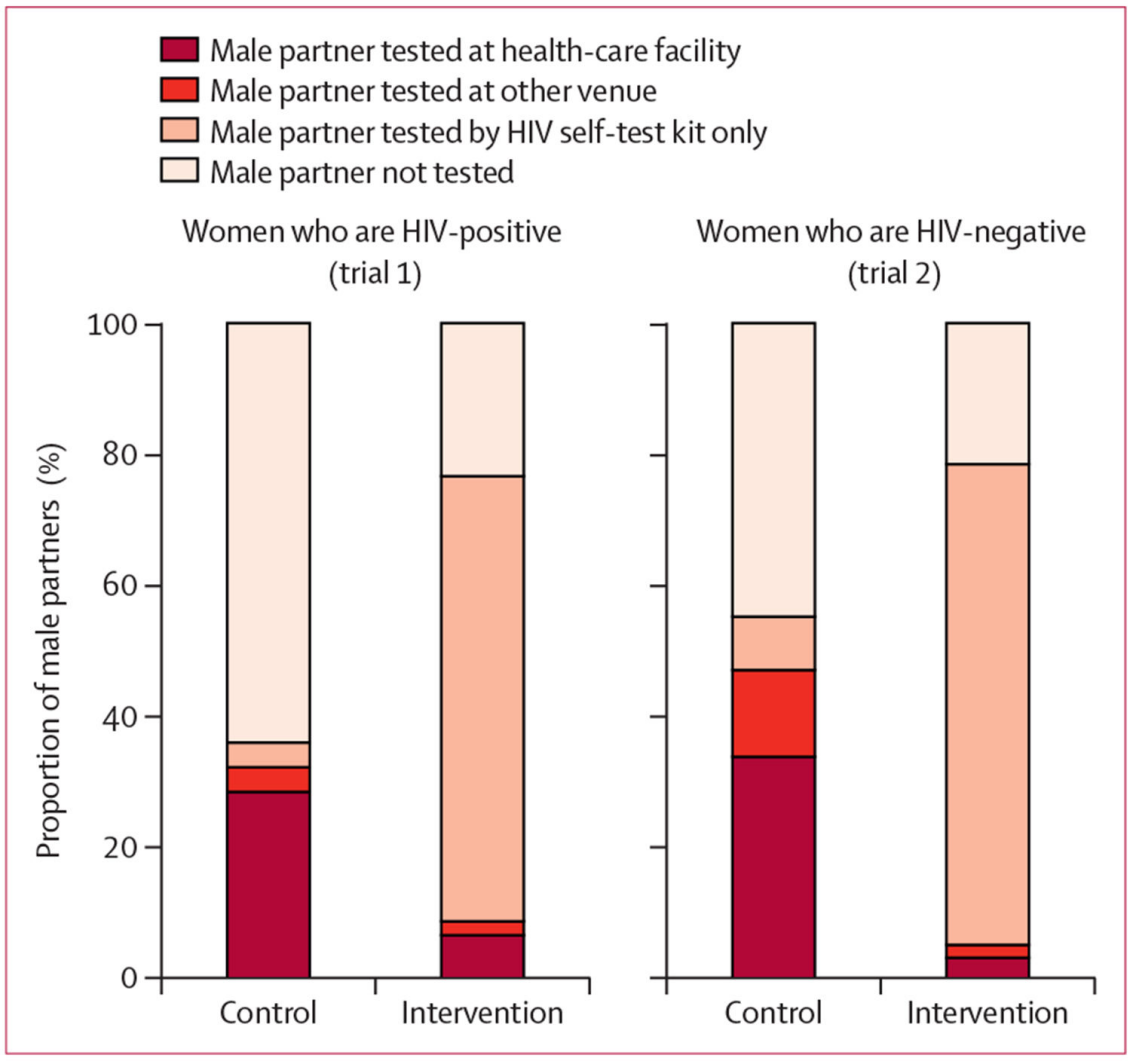
Type of male partner HIV testing by trial and by randomisation group The darkest red portion of the bar represents the primary endpoint (male partner HIV testing at a health facility). The darkest three portions of the bar together represent an additional prespecified endpoint (male partner HIV testing of any kind).

**Figure 3:**
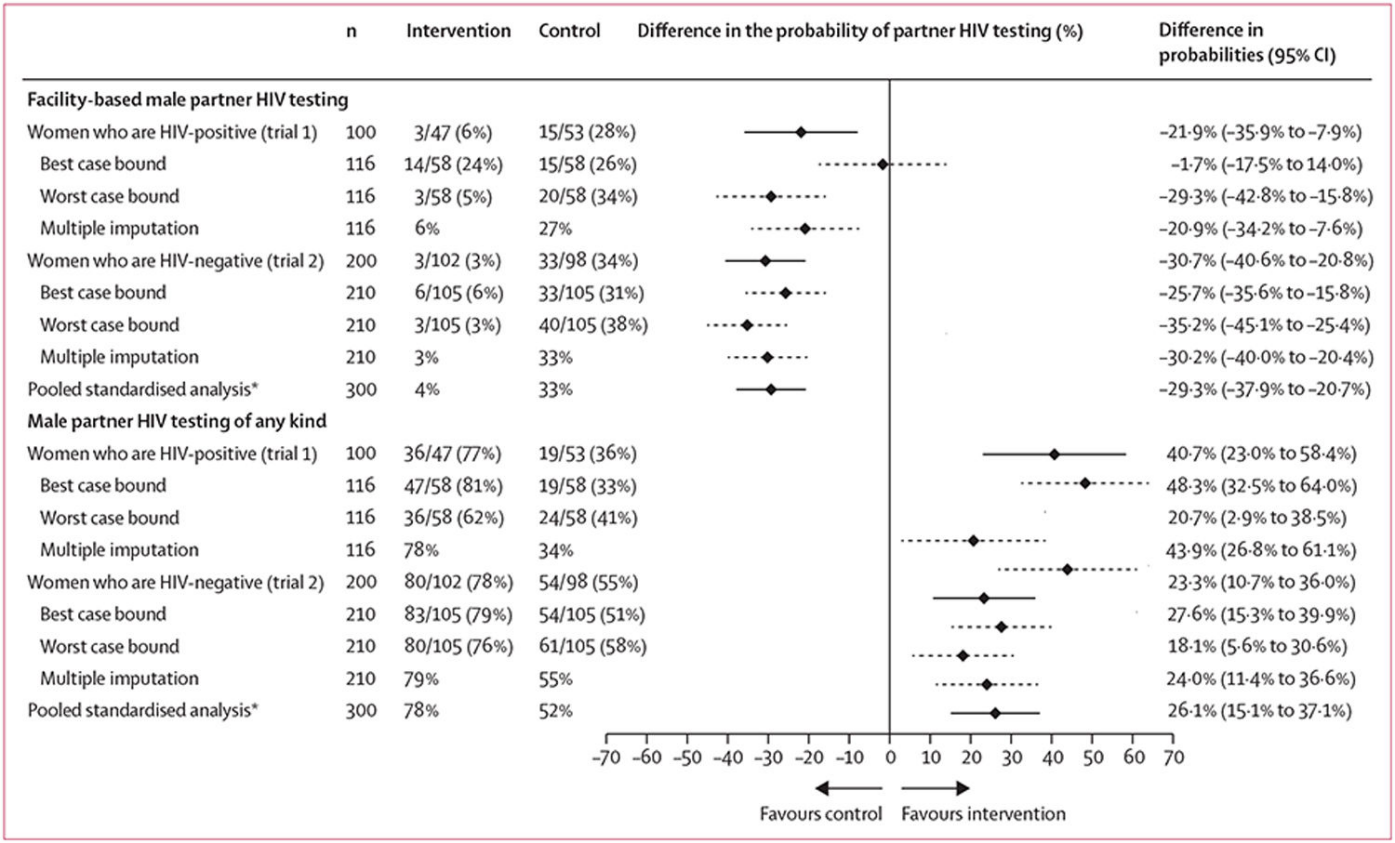
Unadjusted probability difference estimates of HIV testing uptake in male partners of enrolled pregnant women *In the pooled standardised analyses, the two trials are weighted by antenatal HIV prevalence at the study site.

**Figure 4:**
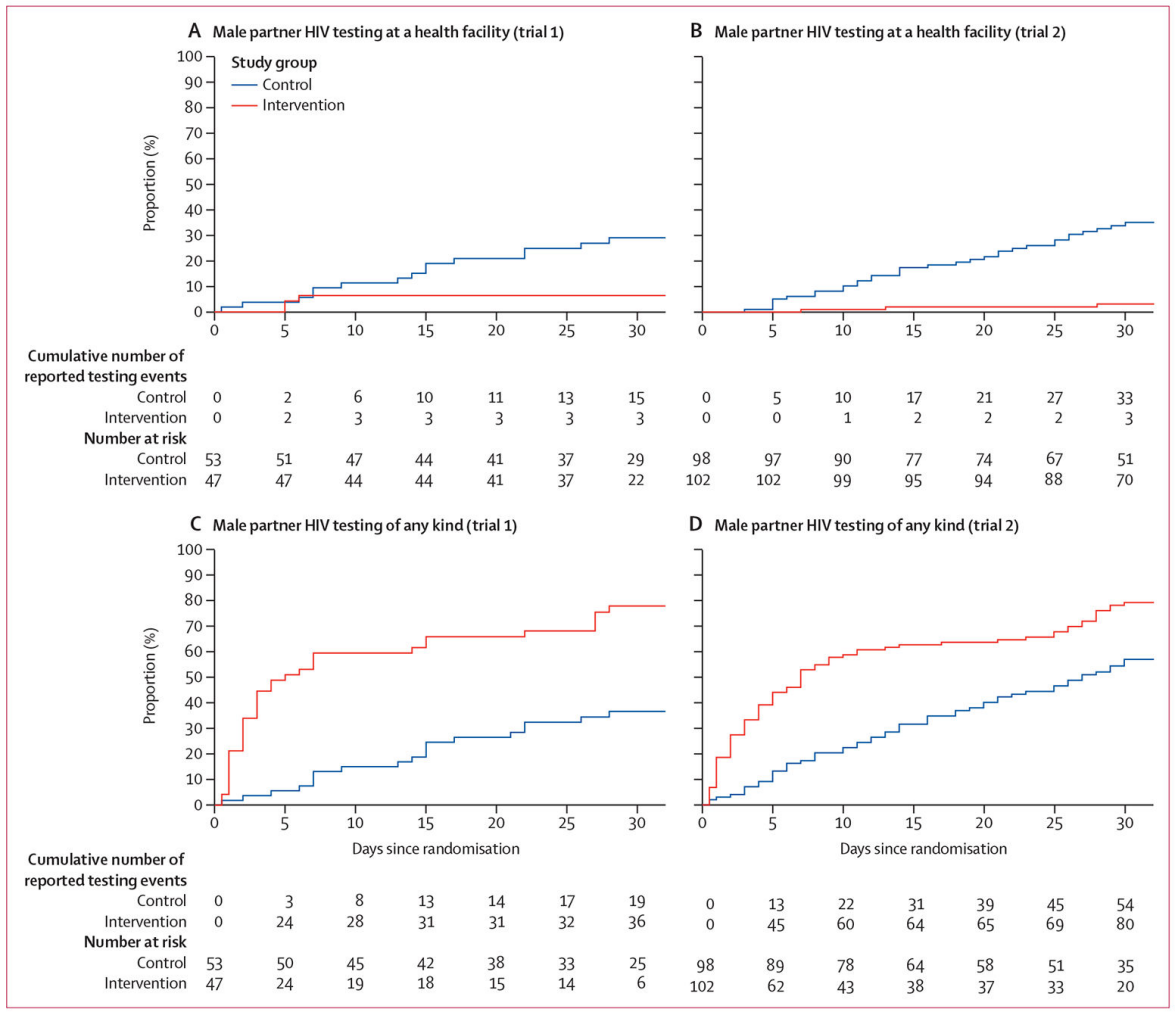
Kaplan-Meier graphs of reported male partner HIV testing over the 30 days following randomisation The two top figures show the control and intervention groups in trial 1 (A) and trial 2 (B) for male partner HIV testing at a health facility. The bottom two figures show the control and intervention groups in trial 1 (C) and trial 2 (D) for male partner HIV testing of any kind.

Reported male partner HIV testing methods, stratified by trial and randomisation group, are shown in figure 2 and table 2. In trial 1, 47 (81%) of 58 women randomly assigned to the intervention group, and 53 (92%) of 58 women randomly assigned to the control group returned for a follow-up visit. In trial 2, 102 (97%) of 105 of women randomly assigned to the intervention group and 98 (93%) of 105 women randomly assigned to the control group returned for a follow-up visit. Across both trials, those allocated to the intervention group were less likely to report male partner facility-based HIV testing within 30 days (our primary outcome). Of the women who were HIV-positive, 3 (6%) of 47 in the intervention group versus 15 (28%) of 53 in the control group reported facility-based male partner HIV testing (estimated probability difference −21·9 [95% CI −35·9 to −7·9]). Of the women who were HIV-negative, 3 (3%) of 102 in the intervention group versus 33 (34%) of 98 in the control group reported facility-based male partner HIV testing (estimated probability difference −30·7% [95% CI −40·6% to −20·8%]; figure 3). Sensitivity bounds for the best and worst case for missing data supported the direction of effect for both outcomes; multiple imputation results were similar to the complete-case analysis as well. In pooled standardised analysis accounting for antenatal HIV prevalence at the study site, the estimated probability difference was −29·3% (95% CI −37·9% to −20·7%). Kaplan-Meier estimates of the time to male partner HIV test are shown in figure 4. Results from adjusted analyses were similar to the unadjusted results (appendix).

When we assessed an additional prespecified outcome—HIV testing of any kind within 30 days of randomisation—the opposite effect was observed. Of the women who were HIV-positive, those randomly assigned to the intervention group were more likely to report male partner HIV testing of any kind than the control group (77% *vs* 36%; estimated probability difference 40·7% [95% CI 23·0–58·4]). A similar, but smaller, difference was noted in the women who were HIV-negative: male partner HIV testing of any kind was more frequently reported in the intervention group than the control group (78% *vs* 55%; estimated probability difference 23·3% [95% CI 10·7–36·0]; figure 3). In pooled standardised analysis, the estimated probability difference was 26·1% (95% CI 15·1–37·1).

**Figure 5:**
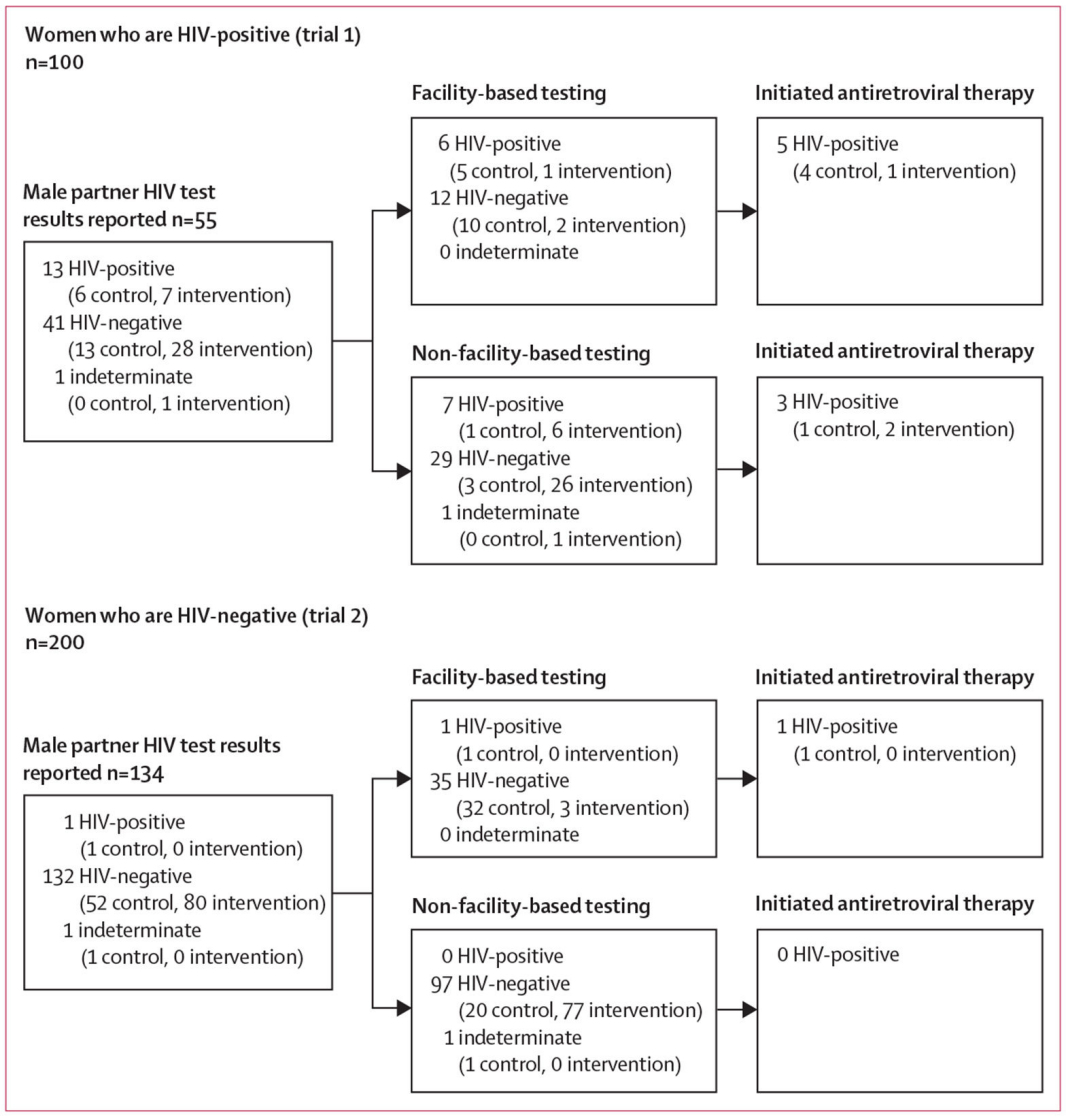
Flow diagram showing reported male partner HIV test results and subsequent linkage to care for those who were diagnosed with HIV

16 of the 116 HIV-positive women enrolled in trial 1 did not report the primary outcome, and of the 100 who did, 55 (55%) reported that their male partners had been tested for HIV. Of these, 13 (68%) of 19 male partners in the control group and 28 (78%) of 36 male partners in the intervention group were reported to be HIV-negative in HIV serodiscordant relationships. 13 (24%) of the 55 male partners had an HIV-positive result, of whom eight were reported to be taking ART. In trial 2, ten of 210 HIV-negative women enrolled did not report the primary outcome, and among the 200 who did, 134 (67%) reported that their male partners had been tested for HIV. The vast majority of these male partners tested HIV-negative: 52 (96%) of 54 male partners in the control group and all 80 (100%) in the intervention group. In the control group, one male partner received an indeterminate result and one received a positive HIV result; the latter received HIV testing at a health-care facility and was reported to be taking antiretroviral therapy. These findings are detailed in figure 5.

Social harms were infrequently observed throughout the study. One HIV-positive woman (control) reported intimate partner violence, male partner abandonment, and emotional and legal harm arising from study participation. Two other women who were HIV-positive (one intervention and one control) reported intimate partner violence, but did not link these events to their participation in this study. No women in trial 2 reported social harms or intimate partner violence. One maternal death occurred over the course of the study: a participant in trial 1 who had been allocated to the intervention group. She was diagnosed with meningoencephalitis due to advanced HIV disease, a condition deemed unrelated to study participation.

## Discussion

We compared strategies for HIV testing of male partners of pregnant women who are HIV-positive and pregnant women who are HIV-negative in antenatal settings: partner notification plus secondary distribution of HIV self-test kits (intervention) versus partner notification alone (control). Our integrated intervention was associated with decreases in facility-based HIV testing and increases in HIV testing of any kind (driven by self-testing) in male partners of all women irrespective of their HIV status. Such combination strategies can help to expand male partner HIV testing out of antenatal settings. However, stronger supporting services might be needed to link specific groups (eg, men who are HIV-positive and men at high risk for HIV acquisition) to health services.

To reach our primary endpoint, male partners of pregnant women had to undergo HIV testing at a health facility. We designated this as our primary outcome for several reasons. First, facility-based HIV testing services present important opportunities for education, couples-based counselling, and engagement with long-term care. For men who test positive for HIV using a self-test kit, the need for confirmatory HIV testing is evident. At the time of our study, HIV testing services also provided one of the few entry points into nascent HIV prevention programmes, including those for HIV pre-exposure prophylaxis. As such, we considered facility-based HIV testing as a reasonable proxy for engagement with comprehensive HIV services. Second, from a study design perspective, we sought a standard outcome that could be used across both trials, irrespective of the male partner’s HIV test result. Because WHO recommends facility-based HIV testing for key groups (eg, men who test positive using a self-test kit and men who test negative for HIV using a self-test kit but are at ongoing risk of HIV), we applied the same standard for all male partners. Finally, we recognise that this strict primary outcome might not fully align with the latest WHO guidelines for low-risk individuals who test negative for HIV using a self-test kit.^11^ However, from a research perspective, it provides novel insights into male partner HIV testing behaviours. As HIV programmes in Lusaka, Zambia continue to evolve and incorporate an increasing number of facets of differentiated and decentralised care, other outcomes might become increasingly programmatically relevant in the future.

Over the course of study implementation, new approaches for HIV testing, including HIV self-testing and community-based testing,^22,23^ expanded across our hospital catchment area. To better contextualise our findings within this changing programmatic landscape, we added a complementary outcome (ie, HIV testing of any kind) in prespecified analyses. By considering both facility-based male partner HIV testing versus male partner HIV testing of any kind, our analysis provides a nuanced view about the potential effect of adding HIV self-testing. On one hand, when HIV self-testing is offered in addition to partner notification, the proportion of male partners who undergo HIV testing increases substantially. On the other hand, relocating HIV testing outside of the health-care infrastructure can create lost opportunities to engage with existing HIV services. This latter explanation is evident in our results, in which women in the intervention groups were less likely to report that their male partners accessed facility-based HIV testing services than those in the control groups. Although features of our study design could have contributed to these findings, including the short follow-up window of 30 days, our results appear consistent with other recent studies.^17,24^

Our findings suggest that adding HIV self-testing to partner notification services can expand the coverage of male partner HIV testing and help to identify those in immediate need of HIV prevention or treatment. To fully realise this potential, however, stronger linkages to care are needed between communities and health facilities. For individuals living with HIV, efforts to promote linkages between HIV diagnosis and treatment have garnered increasing attention;^25^ however, as evident from our results, gaps might remain. In the context of HIV prevention, such linkages could present further challenges. A proportion of male partners who test HIV-negative could still be at elevated risk for acquiring HIV: of the 55 women who were HIV-positive whose partner received HIV testing in our study, for example, 41 (75%) stated that their partners had tested HIV-negative; a surprisingly high rate of HIV serodiscordancy. Because many national programmes do not recommend facility-based, confirmatory HIV testing when HIV self-test kits are non-reactive (including in Zambia^14^), alternative venues might be needed to link men to comprehensive prevention services, including HIV pre-exposure prophylaxis.

We observed a low incidence of reported intimate partner violence and social harms. This could be attributed in part to our eligibility criteria for the study, which excluded those at high risk of partner violence or social harms. However, our findings did not differ by study group and are consistent with other studies of HIV self-testing of male partners.^15,26^ Nevertheless, strategies relying on the secondary distribution of HIV self-test kits can place an undue burden on pregnant and breastfeeding women.^27^ Given the challenges inherent to HIV status disclosure, resources are needed to minimise intimate partner violence and social harms, and actively support those who face such issues.^27^

Our study has numerous strengths, including its randomised design and broad eligibility criteria. Although we did separate trials for HIV-positive and HIV-negative pregnant women, our pooled standardised analysis showed consistent population-level effects when accounting for HIV prevalence. We also note several limitations. First, we relied on participant self-report for our main outcomes and this could result in recall and reporting biases. With growing access to community-based HIV testing,^22,28^ male partners might have tested without the index participant’s knowledge. It is also possible that participants responded in ways they felt were socially desirable in the health-care setting. Second, allocation to study groups was not masked. Participants were aware of their study group assignment and this could have influenced their subsequent health behaviours. Third, because of the importance of male partner engagement throughout pregnancy, we used a short window (ie, 30 days) for our outcome measures. It is possible that an increased uptake of male partner HIV testing could have been reported if we had extended the partner testing window beyond 30 days. Fourth, with the emergence of the COVID-19 pandemic in March 2020, public health measures implemented by the Zambian Government limited our ability to trace participants for missed visits. We did numerous sensitivity analyses to account for follow-up losses and these results were largely in agreement with our complete-case approach. Hospital-level mitigation strategies (eg, reduced clinical staff and restrictions on accompanying family members) could have also reduced access to HIV testing services, but rates of reported facility-based HIV testing did not substantially change over this period. Fifth, the partner notification component, which was part of both the control and intervention groups, included four different options, in line with WHO recommendations and Zambia HIV guidelines. Across both trials, the overwhelming majority of participants selected client selfreferral, which is a passive approach. In settings in which other options are preferred or more accessible, such comparisons might yield different results. Finally, this report focuses on the comparative outcomes in male partner HIV testing. Further analyses are underway to describe the feasibility and acceptability of the intervention, as well as its effect on couples HIV counselling and testing.

In summary, our results show both the opportunities and the challenges inherent to this integrated strategy for male partner HIV testing. HIV self-testing and partner notification services increased male partner testing but reduced the proportion of men seeking facility-based HIV testing. As national HIV programmes seek to meet the ambitious 95–95–95 goals set by UNAIDS for 2030,^29^ such integrated strategies can play an important role, and optimised linkages are needed to ensure that all men who undergo HIV testing receive timely access to comprehensive HIV prevention and treatment.

## Supplementary Material

1

## Figures and Tables

**Table 1:** Baseline characteristics of pregnant women enrolled in the two parallel randomised trials

	Women who were HIV- positive (trial 1)		Women who were HIV-negative (trial 2)	
	Control group (n=58)	Intervention group (n=58)	Control group (n=105)	Intervention group (n=105)
Age at enrolment, years	26 (23–29)	26 (23–30)	25 (22–28)	24 (21–28)
Primary school complete				
Yes	41 (71%)	36 (62%)	76 (72%)	77 (73%)
No	17 (29%)	22 (38%)	29 (28%)	28 (27%)
Household characteristics				
No electricity or running water	8 (14%)	14 (24%)	15 (14%)	13 (12%)
Electricity only	32 (55%)	23 (40%)	63 (60%)	58 (55%)
Running water only	0	1 (2%)	4 (4%)	3 (3%)
Both electricity and running water	18 (31%)	20 (34%)	23 (22%)	31 (30%)
Travel time to clinic, min				
<30	11 (19%)	17 (29%)	25 (24%)	23 (22%)
30–59	32 (55%)	28 (48%)	56 (53%)	62 (59%)
≥60	15 (26%)	13 (22%)	24 (23%)	20 (19%)
Gestational age at enrolment, weeks	24 (20–28)	24 (20–28)	28 (24–32)	28 (24–32)
Number of pregnancies (including current pregnancy)	3 (1–3)	3 (2–4)	2 (2–3)	2 (1–3)
Primigravida	16 (28%)	9 (16%)	26 (25%)	37 (35%)
Number of living children	1 (0–2)	1 (0–2)	1 (0–2)	1 (0–2)
ART use				
ART naive	26 (45%)	24 (41%)	NA	NA
Current ART user	31 (53%)	34 (59%)	NA	NA
Prior ART user	1 (2%)	0	NA	NA
Ever consumed alcohol				
Yes	5 (9%)	8 (14%)	9 (9%)	15 (14%)
No	53 (91%)	50 (86%)	96 (91%)	90 (86%)
Intimate partner violence in past 12 months				
Yes	1 (2%)	4 (7%)	1 (1%)	1 (1%)
No	57 (98%)	54 (93%)	104 (99%)	104 (99%)
Number of lifetime male sex partners				
1	16 (28%)	8 (14%)	46 (44%)	46 (44%)
2–3	37 (64%)	39 (67%)	52 (50%)	45 (43%)
≥4	5 (9%)	11 (19%)	7 (7%)	14 (13%)
Multiple partners in the past 6 months				
Yes	1 (2%)	2 (3%)	0	0
No	57 (98%)	56 (97%)	105 (100%)	105 (100%)
Primary partner’s age, years^*^	31 (27–35)	32 (29–38)	30 (27–34)	29 (27–35)
Age difference between woman and primary partner, years^*^	5 (3–7)	5 (3–9)	4 (3–8)	5 (4–7)
Length of relationship with primary partner, years	3 (2–6)	3 (1–6)	5 (2–8)	4 (1–8)
Resides with primary partner				
Yes	49 (84%)	49 (84%)	102 (97%)	89 (85%)
No	9 (16%)	9 (16%)	3 (3%)	16 (15%)
Married to primary partner				
Yes	51 (88%)	52 (90%)	103 (98%)	92 (88%)
No	7 (12%)	6 (10%)	2 (2%)	13 (12%)
Number of sexual intercourse acts with primary partner in the past 30 days	6 (3–12)	8 (3–12)	8 (4–12)	6 (3–12)
No sexual intercourse acts in past 30 days	9 (16%)	5 (9%)	6 (6%)	15 (14%)
Consistent condom use with primary partner in past 30 days†				
Yes	4/49 (8%)	2/53 (4%)	3/99 (3%)	1/90 (1%)
No	45/49 (92%)	51/53 (96%)	96/99 (97%)	89/90 (99%)
Disclosed current HIV status to primary sex partner‡				
Yes	30/57 (53%)	37/58 (64%)	103/104 (99%)	98/105 (93%)
No	27/57 (47%)	21/58 (36%)	1/104 (1%)	7/105 (7%)
Primary partner HIV testing history				
Never tested	24 (41%)	17 (29%)	40 (38%)	41 (39%)
Previously tested	22 (38%)	22 (38%)	46 (44%)	35 (33%)
Unknown	12 (21%)	19 (33%)	19 (18%)	29 (28%)
Primary partner used HIV self-testing kit at last HIV test§				
Yes	1/22 (5%)	0/22	2/46 (4%)	2/34 (6%)
No or don’t know	21/22 (95%)	22/22 (100%)	44/46 (96%)	32/34 (94%)
Received couple HIV testing and counselling with primary partner before current pregnancy‡				
Yes	2/22 (9%)	1/22 (5%)	2/46 (4%)	2/35 (6%)
No	20/22 (91%)	21/22 (95%)	44/46 (96%)	33/35 (94%)

Data are median (IQR) or n (%). ART=antiretroviral therapy. NA=Not applicable.

* Two participants in trial 2 reported unknown partner age.

† Only includes participants who reported at least one sexual intercourse act in the past 30 days.

‡ Two participants (one in the control group of trial 1 and one in the control group of trial 2) did not provide a response to this question.

§ Only includes participants who reported their primary partner had been previously tested for HIV. One participant (trial 2, intervention) did not provide a response to this question.

**Table 2:** Type of male partner HIV testing by trial and by randomisation group

	Women who are HIV-positive (trial 1)		Women who are HIV-negative (trial 2)	
	Control (n=53)	Intervention (n=47)	Control (n=98)	Intervention (n=102)
Male partner tested at health-care facility	15 (28%)	3 (6%)	33 (34%)	3 (3%)
Male partner tested at other venue	2 (4%)	1 (2%)	13 (13%)	2 (2%)
Male partner tested by HIV self-test kit only	2 (4%)	32 (68%)	8 (8%)	75 (74%)
Male partner not tested	34 (64%)	11 (23%)	44 (45%)	22 (22%)

## Research in context

### Evidence before this study

The promotion of male partner involvement in programmes for the prevention of mother-to-child (PMTCT) HIV transmission has been shown to improve maternal and child outcomes. Testing male partners for HIV is an important aspect of such involvement, offering entry to family-centred care and guiding HIV prevention and treatment interventions. Several single-modality strategies (eg, partner notification, HIV self-testing, and home-based HIV testing) are associated with moderate increases in male partner HIV testing. Approaches that combine one or more of these evidence-based practices might further enhance male partner HIV testing rates and deserve further study. We searched PubMed on Dec 2, 2020, for randomised trials to increase male partner HIV testing in antenatal settings using the following search terms: “HIV test” AND “partner” AND “antenatal OR postnatal” AND “randomised”. There were no date restrictions on our search, and we restricted results to those in English. Overall, 39 articles were identified, with only 16 focused primarily on male partner HIV testing. Results from five interventional trials were reported; two of these described combination strategies to increase male partner testing in antenatal settings. In Kenya, the provision of HIV self-test kits and an enhanced invitation card resulted in higher proportions of male partner HIV testing (334 [79%] of 422), than an enhanced invitation alone (136 [35%] of 387) or the standard Ministry of Health invitation card (110 [27%] of 406; p<0·001. A cluster-randomised trial in Malawi found that four different combination strategies, all incorporating HIV self-testing, showed higher levels of male partner HIV testing (87% to 95%) than the standard invitation letter to encourage male partner HIV testing (71 [17%] of 408, p<0·001). Both studies were inclusive of all pregnant women, regardless of their HIV status, but neither reported testing outcomes or adverse events, stratified by the woman’s HIV status.

### Added value of this study

We compared two different approaches to increase male partner HIV testing in antenatal settings based on strategies recommended by WHO. In the control groups, women received a single strategy: partner notification services. In the intervention groups, women received a combination of partner notification services and secondary distribution of HIV self-test kits. In contrast to previous trials, both study groups included assisted methods to increase male partner HIV testing. In parallel trials, which separately enrolled HIV-positive and HIV-negative pregnant women, the addition of HIV self-testing decreased reported facility-based testing (our primary outcome) by 22% among male partners of women who were HIV-positive and by 31% among male partners of women who were HIV-negative. At the same time, the proportion who underwent HIV testing of any kind increased by 41% among partners of women who were HIV-positive and 23% among partners of women who were HIV-negative, due to increased use of HIV self-testing. Our study provides a nuanced look at male partner HIV testing, including preferences in approach and venue. It also offers important insights about HIV testing in male partners of women who were HIV-negative, a group that is often overlooked but contributes to new maternal, and subsequent infant, HIV infections.

### Implications of all the available evidence

When combined with partner notification, secondary distribution of HIV self-test kits can increase male partner HIV testing in antenatal settings. To fully deliver on the promise of combination male partner HIV strategies, however, additional research is needed to enhance linkages (from HIV testing to formal HIV prevention and treatment programmes) so that recently tested male partners are appropriately connected to the health services they need.
