# Supplementary material for: Addition of HIV self-test kits to partner notification services to increase HIV testing of male partners of pregnant women in Zambia: two parallel randomised trials
Source: Lancet Glob Health. Author manuscript; Available in PMC 2021 Dec 5. (PMC8644317; doi:10.1016/S2214-109X(21)00393-4)
Supplement: 1 [file NIHMS1757890-supplement-1.pdf]

THE LANCET
Global Health

Supplementary appendix

This appendix formed part of the original submission and has been peer reviewed. We post it as supplied by the authors.

Supplement to: Mutale W, Freeborn K, Graybill LA, et al. Addition of HIV self-test kits to partner notification services to increase HIV testing of male partners of pregnant women in Zambia: two parallel randomised trials. *Lancet Glob Health* 2021; published online Nov 1. http://dx.doi.org/10.1016/S2214-109X(21)00393-4.

Appendix Table 1: Adjusted estimates of HIV testing uptake among male partners of enrolled pregnant women

	n	Intervention group: Potential Outcome Probability	Control group: Potential Outcome Probability	Adjusted Difference in Probabilities (95% CI)†
**Facility-based male partner HIV testing reported**				
Trial 1: HIV Positive Women	100	5·5%	26·4%	-21·0% (-33·9%, -7·5%)
Worst case bound	116	4·3%	34·1%	-29·7% (-43·4%, -15·1%)
Best case bound	116	23·5%	25·9%	-2·4% (-18·0%, 12·7%)
Trial 2: HIV Negative Women*	198	3·1%	34·5%	-31·4% (-42·9%, -21·9%)
Worst case bound	208	3·0%	39·2%	-36·2% (-47·3%, -26·9%)
Best case bound	208	5·5%	32·4%	-26·9% (-37·1%, -17·1%)
**Male partner testing of any kind reported**				
Trial 1: HIV Positive Women	100	79·3%	34·3%	45·0% (27·6%, 60·3%)
Worst case bound	116	63·7%	41·6%	22·1% (3·8%, 40·1%)
Best case bound	116	83·2%	33·6%	49·6% (33·9%, 63·5%)
Trial 2: HIV Negative Women*	198	78·4%	56·2%	22·3% (9·4%, 35·5%)
Worst case bound	208	76·4%	59·2%	17·2% (4·2%, 29·6%)
Best case bound	208	79·0%	52·4%	26·5% (14·1%, 38·9%)

All models adjusted for: partner HIV testing history (ever tested vs. never tested/unknown), partner age (in years, continuous), age difference between couple (in years, male minus female age, continuous), and travel time to clinic (<30, 30-59, 60+ minutes).

* Two participants in Trial 2 are missing information on partner age. Total sample size for complete case analysis is therefore 198, while total sample size for sensitivity analyses is therefore 208.

† Bias-corrected percentile bootstrapped 95% CI.
